# Facing the challenge of multimorbidity*

**DOI:** 10.15256/joc.2016.6.71

**Published:** 2016-02-17

**Authors:** Boris Azaïs, John Bowis, Matthias Wismar

**Affiliations:** ^1^Public Policy Europe & Canada, MSD, Courbevoie, France; ^2^Health First Europe, Brussels, Belgium; ^3^European Observatory on Health Systems and Policies, Brussels, Belgium; and WHO European Centre for Health Policy, Brussels, Belgium

**Keywords:** multimorbidity, multiple chronic conditions, comorbidity, European Health Forum Gastein, public policy, health systems

Multimorbidity is a major public health challenge that is rising up the political and health agenda at an accelerated rate. Although the prevalence of multimorbidity increases with age, more than half of the population with multimorbidity are under the age of 65 years [1], with social deprivation a key determinant of multimorbidity in young and middle-aged adults [2, 3].

From an individual’s perspective, multimorbidity reduces life expectancy [4–6], decreases physical functioning and quality of life [7], and increases the risk of depression and other mental health disorders [3]. From a healthcare provider’s perspective, multimorbidity is associated with increased health service use, a high risk of emergency and other hospital admissions, high rates of polypharmacy, and spiralling costs [8]. Current health systems, which are typically built around a single-disease framework, are poorly adapted to cope with patients with multimorbidity, who typically experience fragmented healthcare services, leading to potentially inefficient and ineffective care.

It is increasingly clear that we need to change our perspective on multimorbidity in order to address it as a specific condition that requires tailored solutions and approaches. The urgent need to tackle multimorbidity in a more strategic, holistic, and cost-effective manner was evident at the 18th European Health Forum Gastein, a leading annual health policy event in the European Union (EU), held in the autumn of 2015. This Forum attracted policymakers, clinicians, health service managers, patients, and a broad range of other stakeholders, all of whom were invited to attend a session entitled “*Facing the Challenge of Multimorbidity*”, which was supported by an unrestricted educational grant from MSD (a subsidiary of Merck & Co., Inc., NJ, USA). The session included presentations from many of Europe’s most influential policymakers, researchers, and campaigners on the issue of multimorbidity, and sparked lively discussions and debate.

This supplement to the *Journal of Comorbidity* summarizes the presentations and discussions from this important meeting, and outlines many of the key challenges we are currently facing in the management of multimorbidity. In the first paper [9], the epidemiology, risk factors, and consequences of multimorbidity are reviewed, highlighting how relatively little we actually know about multimorbidity and its determinants, or the effectiveness or cost-effectiveness of current interventions. This lack of basic knowledge will continue to hamper the efforts of policymakers; however, work is already underway to fill some of these information gaps, and this paper outlines how the European Commission and other public bodies are contributing to this effort.

The second paper in the supplement [10] focuses on different models of care and how they might be applied to people with multimorbidity to improve outcomes. Under current silo-based models, patients with multimorbidity are forced to navigate between multiple care providers who may have conflicting clinical priorities and who may not communicate well with each other. In these circumstances, care coordination becomes a challenge, and patients can be left feeling overwhelmed and under-supported. This paper by Albreht *et al*. describes new integrated models of care that have been developed specifically for people with multimorbidity, and discusses the importance of disease prevention, patient-centred care delivery, multidisciplinary teamwork, and shared decision-making within these models.

In the third paper [11], three influential economists and policymakers review where we are today in terms of developing more sustainable health systems to tackle multimorbidity, and what may still be required to reduce the burden of disease. Again, a lack of basic knowledge about multimorbidity is highlighted by these experts, but it is clear from their review that there is a pressing need to strengthen primary care and develop innovative financing and delivery systems that measure and reward both quality and performance.

Finally, the issue of patient experience of care is explored in the fourth paper [12], which has been co-authored by people whose lives have been touched by multimorbidity. From a patient perspective, it would seem that being knowledgeable, experiencing good care coordination and communication, and being involved in the decision-making all contribute towards a better experience of care. The authors highlight the importance of integrating physical health, mental health, and social care, of simplifying care pathways, and of improving equitable access to quality care for all. Politicians and health service providers are urged by these authors to incorporate the views of patients when developing future policies on multimorbidity.

As can be seen from the articles in this special issue, we have reached an important milestone in the management of patients with multimorbidity. We have recognized that major reforms are needed, and we have identified key areas where more work is required. We have broadly agreed that greater investment is needed in multimorbidity prevention, in patient education for self-management, and in strengthening primary care. We understand that our health systems need to be reshaped to deliver more patient-centred, integrated care, where multidisciplinary teamwork is standard and the patient experience is a concrete measure of care quality. We have seen how new integrated care models have begun to deliver positive results for patients and healthcare systems, and how incentivizing providers to manage patients in a more holistic way may benefit patients with multimorbidity in the future.

The World Health Organization, the European Commission, governments, policymakers, and care providers across Europe are at last facing the challenge of multimorbidity, and we look forward to hearing further news of the many different collaborations, initiatives, programmes, and projects underway to improve the management of multimorbidity in Europe. We are proud to have been part of such a dynamic and informative event, and are confident that there will be plenty of interest for all involved in the care of patients with multimorbidity within the pages of this supplement.
